# Diverse impact of acute and long-term extracellular proteolytic activity on plasticity of neuronal excitability

**DOI:** 10.3389/fncel.2015.00313

**Published:** 2015-08-10

**Authors:** Tomasz Wójtowicz, Patrycja Brzdąk, Jerzy W. Mozrzymas

**Affiliations:** ^1^Laboratory of Neuroscience, Department of Biophysics, Wroclaw Medical UniversityWroclaw, Poland; ^2^Department of Animal Physiology, Institute of Experimental Biology, Wroclaw UniversityWroclaw, Poland

**Keywords:** extracellular proteases, intrinsic excitability, E-S potentiation, plasticity, LTP, hippocampus

## Abstract

Learning and memory require alteration in number and strength of existing synaptic connections. Extracellular proteolysis within the synapses has been shown to play a pivotal role in synaptic plasticity by determining synapse structure, function, and number. Although synaptic plasticity of excitatory synapses is generally acknowledged to play a crucial role in formation of memory traces, some components of neural plasticity are reflected by nonsynaptic changes. Since information in neural networks is ultimately conveyed with action potentials, scaling of neuronal excitability could significantly enhance or dampen the outcome of dendritic integration, boost neuronal information storage capacity and ultimately learning. However, the underlying mechanism is poorly understood. With this regard, several lines of evidence and our most recent study support a view that activity of extracellular proteases might affect information processing in neuronal networks by affecting targets beyond synapses. Here, we review the most recent studies addressing the impact of extracellular proteolysis on plasticity of neuronal excitability and discuss how enzymatic activity may alter input-output/transfer function of neurons, supporting cognitive processes. Interestingly, extracellular proteolysis may alter intrinsic neuronal excitability and excitation/inhibition balance both rapidly (time of minutes to hours) and in long-term window. Moreover, it appears that by cleavage of extracellular matrix (ECM) constituents, proteases may modulate function of ion channels or alter inhibitory drive and hence facilitate active participation of dendrites and axon initial segments (AISs) in adjusting neuronal input/output function. Altogether, a picture emerges whereby both rapid and long-term extracellular proteolysis may influence some aspects of information processing in neurons, such as initiation of action potential, spike frequency adaptation, properties of action potential and dendritic backpropagation.

## Introduction

Learning and memory require alteration in the number and strength of existing synaptic connections. Functional *in vitro* and *in vivo* studies confirmed that memory traces may be encoded by use-dependent modification of synapses. A hallmark of such synaptic plasticity is the long-term potentiation (LTP) or depression (LTD) which can be evoked by patterned stimulation of afferent fibers at high and low frequency, respectively (Malenka and Bear, 2004). In experimental conditions, LTP and LTD were most extensively studied in hippocampal formation in excitatory, and more recently, in inhibitory connections and exact molecular mechanisms vary depending on the synapses and circuits in which they operate (Malenka and Bear, 2004; Nicoll and Schmitz, 2005; Kullmann and Lamsa, 2007). The last decade brought convincing evidence that indeed memory formation uses the same “repertoire” of functional and molecular synaptic modifications as those previously found to accompany long-term plasticity phenomena (Moser et al., 1998; Sacchetti et al., 2001; Whitlock et al., 2006; Nabavi et al., 2014). In particular, synaptic potentiation following behavioral training and that evoked by high-frequency electrical stimulation in the hippocampus occlude each other (Whitlock et al., 2006) strongly indicating common mechanisms.

In most studies aiming at addressing mechanisms of synaptic plasticity, patterned stimulation was commonly used to evoke LTP or LTD. However, no patterned exogenous stimulation can reproduce a complex pattern of endogenous activity of neuronal networks occurring *in vivo*. In addition, changes solely in synaptic strength of synaptic connections such as LTP alone were found to be insufficient to explain memory formation (Zamanillo et al., 1999; Shimshek et al., 2006). For instance, in some reports hippocampal spatial learning occurred in the absence of synaptic LTP (Jeffery, 1997; Silva et al., 1998; D’hooge and De Deyn, 2001). In fact neurons could significantly enhance the information storage capacity by scaling dendritic and somatic excitability (Poirazi and Mel, 2001; Häusser and Mel, 2003; Polsky et al., 2004) and learning (for review, see Zhang and Linden, 2003). Thus, memory storage may involve multiple levels and could be supported by long-term modifications of neuronal input-output properties by far more complex mechanisms than synaptic plasticity alone. Indeed, in the neuronal network in which, besides synaptic strength, additionally neuron firing rate, firing threshold or gain can be modulated, one would expect substantially larger memory storage and information processing capabilities than in the case of alterations in excitatory synaptic function alone. In their early seminal papers, Bliss and colleagues (Bliss and Gardner-Medwin, 1973; Bliss and Lomo, 1973) noticed that in rabbit dentate gyrus population spike amplitude following tetanically evoked synaptic LTP increased far beyond the probability predicted by increased synaptic input [a phenomenon referred to as excitatory post synaptic potential (EPSP)-to-Spike potentiation, E–S potentiation, Bliss and Gardner-Medwin, 1973; Bliss and Lomo, 1973]. Later, studies at the level of single neurons confirmed existence of E-S potentiation and E-S depression (reviewed in Campanac and Debanne, 2008). Interestingly, although E-S potentiation is often observed following stimulation patterns inducing synaptic LTP, changes solely in neuronal excitability in the absence of synaptic gain were reported indicating that synaptic plasticity and intrinsic neuronal excitability are not tightly coupled or at least do not scale linearly (e.g., Ohno et al., 2006). Consequently, taken the importance of action potential generation in transmission of information in neuronal networks, intrinsic neuronal plasticity has been proposed to reflect one of the cellular correlate of behavioral learning and neuronal homeostasis (reviewed in Zhang and Linden, 2003).

In the recent years, rapid, activity-regulated extracellular proteolysis has been implied as an indispensable factor supporting neuronal plasticity learning and memory. In particular, proteolytic activity within and in the vicinity of the synapses has been shown to play a pivotal role in determining synapse structure, function, and number (Huntley, 2012; Sonderegger and Matsumoto-Miyai, 2014). In particular, extracellular proteases determine structural modification of synapses through various pathways, including proteolysis of the extracellular matrix (ECM) proteins, cell adhesion molecules, and neurotrophic factors. Such proteolysis induces changes in the properties of substrate proteins or releases functional domains (ligands) of the substrate proteins, which activate a signal transduction cascades. Several excellent reviews hold information about mechanism whereby extracellular proteolysis could support synaptic plasticity (Ethell and Ethell, 2007; Agrawal et al., 2008; Huntley, 2012; Tamura et al., 2013; Sonderegger and Matsumoto-Miyai, 2014; Ben Shimon et al., 2015), and therefore we will not extensively review this topic here. Importantly, considering the wide range of proteases and their substrates it seems unlikely that they support solely synaptic plasticity. In addition, although the phenomenon of long-term synaptic potentiation and E-S potentiation was discovered at the same time (Bliss and Lomo, 1973), the latter received less attention and molecular mechanisms remain poorly understood. Most recently, we studied for the first time the impact of matrix metalloproteases (MMPs) activity on E-S potentiation in hippocampal CA3 region (Wójtowicz and Mozrzymas, 2014). We found that inhibition of MMPs had detrimental effect on E-S potentiation induced by various LTP-induction protocols. Most strikingly, the reduction in spike potentiation following inhibition of MMPs was more pronounced than it would be expected from impaired synaptic plasticity alone, suggesting that synaptic and nonsynaptic components are regulated by MMPs separately (Wójtowicz and Mozrzymas, 2014). It is important to bear in mind that synaptic and nonsynaptic mechanisms are tightly functionally coupled and deciphering the information processing requires a thorough consideration of these two elements. The fact that recent findings indicate that both of them can be potently regulated by MMPs, places these enzymes at a very strategic position in regulating signal transduction in the neuronal networks. Therefore, here we review the impact of extracellular proteolysis on neuronal plasticity with particular emphasis on nonsynaptic targets and discuss how these processes ultimately alter input-output/transfer function of neurons, supporting cognitive processes. Due to its pivotal role in learning and memory, we will focus on the hippocampal formation but examples of results obtained from studies on other structures will be also presented.

## Neuronal Excitability Following Certain Forms of Learning and Memory *in Vivo*

Following induction of synaptic plasticity in acute brain slices, a shift in neuronal excitability is often observed (for a review, see Daoudal and Debanne, 2003). In most frequently studied CA1 and CA3 hippocampal regions, significant potentiation of population spike amplitude, beyond that predicted solely by gain in synaptic input, occurs following tetanic stimulation of afferent fibers or by pairing synaptic inputs with antidromic action potentials (Andersen et al., 1980; Abraham et al., 1987; Chavez-Noriega et al., 1990; Jester et al., 1995; Wójtowicz and Mozrzymas, 2014). However, the question arises to what extent E-S potentiation occurs *in vivo* following learning?

Past decades of research brought evidence that certain paradigms of learning are associated with E-S potentiation. Initially, persistence of long-term plasticity of intrinsic properties of cell membranes has been demonstrated in invertebrate models (for review, see Mozzachiodi and Byrne, 2010). Later studies showed the same rule exists in vertebrates (reviewed in Zhang and Linden, 2003). With this regard, eyeblink conditioning has been one of the most widely used model systems to study mechanisms of learning and memory. In this paradigm, auditory or visual stimulus is paired with an aversive, eyeblink-eliciting unconditioned stimulus (e.g., a puff of air to the cornea, mild shock or whisker deflection). When applied repeatedly, association is formed such that a learned blink occurs and precedes the unconditioned stimulus. Such learning paradigm involves both cerebellum and hippocampus and the learning-induced potentiation of hippocampal synapses shares similarities with classically obtained hippocampal LTP with respect to components of molecular machinery involved in plastic changes and changes in synapse morphology (reviewed in Christian and Thompson, 2003). Some authors proposed that this paradigm is an example of declarative explicit learning task (Clark and Squire, 1998). With regard to E-S potentiation, it has been shown that trace eyeblink conditioning in rabbits, rats and mice leads to long-term upregulation of neuronal firing in the CA1 region of the hippocampus. A hallmark of increased neuronal excitability is that it occurs as early as 1 h after behavioral training, peaks 24 h after training, and decays over a period of about 1 week (Disterhoft et al., 1986, reviewed in Christian and Thompson, 2003). Interestingly, changes in synaptic input were not typically observed indicating a pivotal role of E-S coupling in this form of plasticity. While studying classical conditioning in a model of trace fear conditioning, enhanced intrinsic excitability of CA1 pyramidal neurons was described (Kaczorowski and Disterhoft, 2009; Mckay et al., 2009).

More recently, environmental enrichment was used to study naturally occurring changes in synaptic efficacy in the hippocampus that underlie experience-induced modulation of learning and memory in rodents. Environmental manipulations, in particular enriched environment, caused an increase in population spike amplitude in the dentate gyrus *in vivo*, while the effects on synaptic transmission were either absent or highly variable, depending on the exposure pattern to the new environment (Irvine et al., 2006). It has been shown that following learning in that paradigm, neurons exhibited decreased spiking threshold and fired significantly more action potentials while no changes were observed on the level of EPSPs (Malik and Chattarji, 2011). Thus, enriched environment may enhance the synaptic plasticity in CA1 neurons but also it may strongly affect the neuronal spiking (Malik and Chattarji, 2011). In agreement with above data, learning in Morris water maze was used to assess the function of neurons from dorsal hippocampus. In particular, dorsal but not ventral hippocampal CA1 neurons exhibited enhanced excitability in animals which learned the water maze task as compared with those from neurons of control rats (Oh et al., 2003). Finally, in a most recent study, periods of neuronal rhythmic firing in rat barrel cortex pyramidal neurons were shown to trigger long-lasting changes in membrane excitability *in vivo* in the absence of altered synaptic input, membrane resting potential or membrane resistance (Mahon and Charpier, 2012). Altogether a picture emerges that both synaptic and nonsynaptic forms of plasticity are substrates for long term memory and work synergistically (Giese et al., 2001; Debanne et al., 2003; Zhang and Linden, 2003). Importantly, changes in neuronal excitability are learning-specific since they are observed exclusively in animals that learned, but not in pseudoconditioned controls or animals that failed to learn (Moyer et al., 1996; Oh et al., 2003; Song et al., 2012). Consistent with a role for intrinsic plasticity in memory consolidation, learning-specific changes in intrinsic neuronal excitability can also serve a metaplasticity function. Thus, the period of enhanced neuronal excitability would match the period during which animals display enhanced learning. Indeed, in one study, olfactory learning resulted in transient enhancement of hippocampal intrinsic excitability and this resulted in facilitation of acquisition of the hippocampus-dependent Morris water maze task (Zelcer et al., 2006). Thus, intrinsic plasticity would support the learning-induced facilitation of learning.

Studies on E-S potentiation phenomenon in acute brain slices were limited to 2–3 h therefore it is important to ask how long E-S potentiation can last? Scarce *in vivo* data indicate that changes in intrinsic excitability accompanying certain paradigms of learning last beyond several hours. In particular, learning-associated upregulation in neuronal intrinsic excitability was observed up to 7 days following learning but not later (reviewed in Sehgal et al., 2013). Therefore, it has been suggested that because of its limited time span it unlikely to encode the memory itself but rather it facilitates successful memory formation (Sehgal et al., 2013). In the next section, we will discuss the mechanisms that were proposed to underlie E-S potentiation.

## The Major Factors Contributing to Potentiation of Neuronal Excitability

As mentioned above, both synaptic and nonsynaptic forms of plasticity often work synergistically. Therefore, the major difficulty in studying the mechanism of altered neuronal activity, firing rate, firing threshold and gain is to separate intrinsic plasticity effects from those induced by increased excitatory synaptic drive. Plasticity of neuronal firing rate, firing threshold or gain within hippocampal circuits requires several factors that can be roughly divided in two groups. The first group is related to enhanced local or global intrinsic membrane excitability, mediated by changes in the expression level or biophysical properties of ion channels affecting dendritic integration, spike generation, signal propagation in dendrites and the axon, and regulation of plasticity thresholds. The second one has synaptic origin and involves decreased somatic inhibition to excitation ratio resulting in local and effective modulation of neuronal output.

### Plasticity of Intrinsic Membrane Excitability

A decade ago, in a seminal paper, Frick and colleagues reported that long-term synaptic potentiation was accompanied by an enhanced local excitability of pyramidal neuron dendrites (Frick et al., 2004). It is now clear that dendrites are not just passive integrators of excitatory synaptic input but actively participate in information processing (reviewed in Johnston and Narayanan, 2008; Sjöström et al., 2008). In particular dendrites can convey fast regenerative action potential-like events mediated by voltage-activated Na^+^ channels (back propagating action potentials, bAPs) or slower events mediated by voltage-activated Ca^2+^ channels called Ca^2+^ dendritic spikes. The basis for these phenomena is the nonuniform and site specific distribution of several voltage gated channels, including Na^+^ channels, A-type K^+^ potassium channels and T-, R- or L-type Ca^2+^ channels across pyramidal neuron extremities. In particular, depending on channel type, phosphorylation state or inactivation kinetics in axon, soma or dendrites, these voltage gated conductances support different functions (reviewed in Waters et al., 2005; Johnston and Narayanan, 2008, see also “Long-Term Effects of Extracellular Proteolysis on Neuronal Excitability” Section below). Functionally, bAPs may act as retrograde signals to dendritic tree indicating the level of neuronal output and similarly to dendritic spikes enhance Ca^2+^ entry through N-methyl-D-aspartate receptors (NMDARs) following removal of Mg^2+^ block in these receptors and activation of Ca^2+^ voltage gated channels (Stuart and Sakmann, 1994; reviewed in Stuart et al., 1997; Waters et al., 2005). Importantly, change in the number, distribution or activity of various ion channels located throughout the neuron may result in altered intrinsic membrane excitability and neuronal input/output function.

Several mechanisms were suggested to operate concomitantly in rapid neuronal activity-dependent modulation of ion channel properties in dendrites. Importantly, mechanisms involved in synaptic plasticity and plasticity of intrinsic membrane excitability share several effectors. First, similar to synaptic plasticity, raise in Ca^2+^ concentration following activation of NMDARs is necessary for induction of E-S potentiation in hippocampus (Jester et al., 1995; Lu et al., 2000; Daoudal et al., 2002; Wójtowicz and Mozrzymas, 2014). Local Ca^2+^ microdomains may support modulation of properties of ion channels regulating neuronal excitability through mechanisms involving calmodulin, calmodulin-dependent kinase II (CaMKII), phosphatase calcineurin and other intracellular effectors (reviewed in Sjöström et al., 2008). Thus, Ca^2+^ from NMDARs would support E-S potentiation by kinase activation and in parallel LTD of GABAergic transmission (e.g., calcineurin activation, Lu et al., 2000). While protein kinase A and C (PKA, PKC) were shown to be crucial for CA1 region neuronal excitability (Hoffman and Johnston, 1998; Yuan et al., 2002), the role of CaMKII was proposed to be negligible (Ohno et al., 2006). A second mechanism involves Ca^2+^-dependent gene expression involving cAMP response element binding protein (CREB) and NF-AT transcription factors. Another mechanism underlying intrinsic plasticity is the regulation of production, trafficking and insertion of ion channels, which results in an altered density of ion channel proteins in the membrane (reviewed in Beck and Yaari, 2008). Finally, activation of receptors of certain neurotransmitters (e.g., acetylcholine, dopamine, serotonin) and activation of metabotropic glutamate and GABA receptors (mGluRs and GABA_B_Rs) was shown to rapidly affect ion channel function through PKC and PKA kinases activity. Channels that were shown to be rapidly modulated through above mentioned mechanisms include e.g., L-type voltage gated Ca^2+^ channels (VGCCs), large-conductance Ca^2+^-activated BK-type K^+^ channels, hyperpolarization activated cyclic nucleotide-gated (HCN) cation-selective channels mediating H-current and many others (reviewed in Sjöström et al., 2008). Thus, following enhanced excitatory synaptic activity, rapid change in channel function may occur with respect to time constants of activation and inactivation, single channel conductance and surface expression and degradation (reviewed in Johnston and Narayanan, 2008; Sjöström et al., 2008). For instance, downregulation of H-current mediated by HCN1/2 channels results in enhanced EPSP summation (reviewed in Beck and Yaari, 2008). In addition, downregulation of A-type K^+^ channels mediating A-current by hyperphosphorylation may result in enhanced bAPs and altered firing (Bernard et al., 2004). In opposite, upregulation of T-type Ca^2+^ channels results in enhanced dendritic Ca^2+^ spikes and altered neuronal firing (Beck and Yaari, 2008).

Besides key aspects of neuronal firing, such as action potential threshold and firing mode, it seems equally important to know how neurons fire repetitively in response to prolonged depolarization. During repetitive firing Ca^2+^-sensitive K^+^ currents are activated which leads to hyperpolarization that lasts tens to hundreds of milliseconds. Afterhyperpolarization (AHP) current affects somatic and dendritic membrane potentials such that by reducing EPSP amplitude in apical dendrites, AHP controls the threshold for neuronal spiking and LTP in CA1 hippocampal region (reviewed in Sehgal et al., 2013). Most importantly, the extent of such AHP determines the rate of repetitive neuronal firing and is downregulated by some learning tasks involving hippocampal formation in intact animals (Disterhoft et al., 1986; Coulter et al., 1989; de Jonge et al., 1990). Moreover, there is a strong and inverse relationship between AHP and magnitude of LTP in acute brain slices: the larger the AHP the smaller the LTP (Sah and Bekkers, 1996; Cohen et al., 1999; Kramar et al., 2004). In addition, age-related learning deficits are reversed with pharmacological manipulations that reduce the normally enlarged post-burst AHP of the CA1 pyramidal neurons observed in aged animals (Disterhoft and Oh, 2006). Most recent work indicates that long-term changes in intrinsic excitability following learning are not only limited to pyramidal neurons, but also hold for interneurons. Learning in the hippocampus-dependent trace eyeblink conditioning task was reported to reduce AHP in somatostatin-positive population of interneurons and enhance inhibition onto CA1 pyramidal neurons (Mckay et al., 2013). Altogether, function of ion channels outside synapses that play an important role in neuronal membrane excitability is regulated following enhanced neuronal activity patterns that lead to neuronal plasticity and formation of memory traces.

### Balance in Somatic Inhibition-Excitation Ratio is Shaping Neuronal Output

The strategic locus of inhibitory drive to principal hippocampal neurons remain soma (Freund and Katona, 2007). By reducing inhibitory tone or responsiveness to inhibitory inputs, neuronal response to integrated excitatory input would increase. Thus, the interplay between excitatory and inhibitory weights remains crucial for basal neuronal input-output function and control of synchrony of principal cell populations (Isaacson and Scanziani, 2011). With regard to neuronal plasticity, changes in the balance between excitation and inhibition (E/I) could directly regulate the plastic potential of neuronal networks. For instance, during cortical development, increasing efficacy of GABAergic inhibition results in progressive reduction in plastic potential of neuronal networks and more extensive hard wiring of existing circuits (Hensch, 2005). Most importantly, invasive or noninvasive (e.g., pharmacological) interventions resulting in decreased strength of certain inhibitory inputs were recently successfully used to enhance experience dependent neuronal plasticity in adult nervous system (reviewed in Bavelier et al., 2010).

Changes in inhibition were proposed to be particularly important for neuronal plasticity following sensory experience. It has been show that in the adult primary auditory cortex, the dynamics of synaptic receptive field plasticity is associated with a reduction of synaptic inhibition which was followed by a large increase in excitation (Froemke et al., 2007). Therefore, in addition to modulation of intrinsic membrane properties, additional important mechanism proposed to underlie E-S potentiation has synaptic origin and involves modulation in somatic inhibition. This view is supported by several studies. For instance, E-S potentiation was completely or partially abolished in the presence of blockers of GABAergic transmission in the hippocampus (Chavez-Noriega et al., 1989; Tomasulo and Ramirez, 1993; Daoudal et al., 2002). Moreover, direct recordings from CA1 pyramidal cells following tetanization of Sch-CA1 projection showed increased excitation/inhibition ratio (Abraham et al., 1987). One proposed candidate for effector mediating suppression of GABAergic inhibition was Ca^2+^-sensitive phosphatase calcineurin, since blockade of this phosphatase prevented induction of E-S potentiation without interfering with synaptic LTP (Lu et al., 2000). Most recently, brief repetitive stimulation of Schaffer collaterals was found to enhance intrinsic neuronal excitability in parvalbumin-positive subpopulation of GABAergic interneurons (PV+ basket cells; Campanac et al., 2013). It was proposed, that following enhanced neuronal activity that results in synaptic plasticity of excitatory synapses, activity of pyramidal neurons could be effectively controlled and balanced by increase in inhibitory input from PV+ basket cells (Campanac et al., 2013). It should be remembered however, that excitatory synaptic transmission onto certain interneurons types also undergoes LTP, keeping the balance between synaptic excitation and inhibition onto principal neurons intact (Lamsa et al., 2005). Moreover, some reports questioned the role of GABAergic inhibition in shaping E-S potentiation (Hess and Gustafsson, 1990; Jester et al., 1995).

## The Role of Extracellular Proteolysis in Shaping Neuronal Excitability

### Acute Actions of Extracellular Proteolysis on Neuronal Spiking

**Figure 1:**
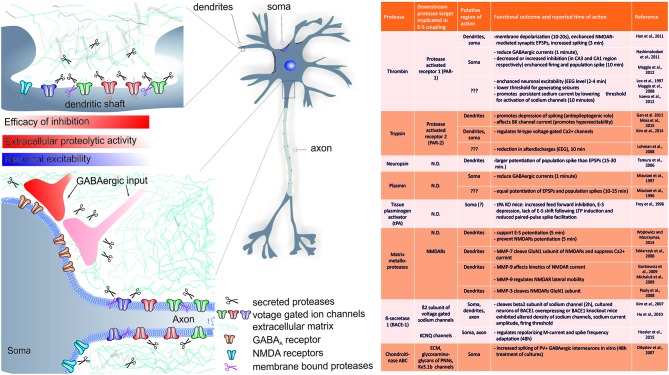
**Summary of the known role of several proteases in rapid (requiring minutes to hours) changes in EPSP-to-spike (E-S) potentiation and/or neuronal spiking**. A cartoon indicates the putative regions of CA1 pyramidal neuron where extracellular proteolysis may affect intrinsic neuronal excitability and E-S potentiation via (a) cleavage of extracellular matrix (ECM) constituents and modulation of ion channels; (b) modulation of inhibitory inputs efficacy (see text for details). The list of known targets of extracellular proteolysis and putative regions of action is listed in the table. N.D.- no data available.

Low levels of several proteases are normally present in hippocampus and include for instance trypsin (Koshikawa et al., 1998), neuropsin (Tomimatsu et al., 2002), neurotrypsin (motopsin, Mitsui et al., 2007), tissue plasminogen activator (tPA; Pawlak and Strickland, 2002), thrombin (Rohatgi et al., 2004) and MMPs (e.g., MMP-2–9, Szklarczyk et al., 2002; Wiera et al., 2012). Activity of these serine proteases and metalloproteases was shown to be crucial for synaptic plasticity and memory (reviewed in Tomimatsu et al., 2002; Shiosaka, 2004; Sonderegger and Matsumoto-Miyai, 2014). Following enhanced neuronal activity such as that occurring during formation of memory traces, proteases may be rapidly released and activated in extracellular space and support synaptic plasticity. For instance, tPA is rapidly secreted following membrane depolarization and Ca^2+^ entry (Gualandris et al., 1996; Parmer et al., 1997; Baranes et al., 1998), while activity of protease neuropsin was shown to rapidly increase as early as 5 min following neuronal activity and activation of NMDARs (Matsumoto-Miyai et al., 2003). Similarly, neurotrypsin may be exocytosed as early as 30 s–2 min post stimulation of neurons with KCl and then removed within several minutes (Frischknecht et al., 2008). In addition, matrix metalloprotease 9 (MMP-9) was shown to follow vesicular release (Sbai et al., 2008) and its activity was detected as early as 5 min following stimulation of cultured neurons with glutamate or blockers of inhibitory transmission (Michaluk et al., 2007). More recently, rapid release and enhanced activity of MMP-9 in the synapse was shown to occur as early as 5–10 min following stimulation (Dziembowska et al., 2012; Stawarski et al., 2014). Thus, taken that use-dependent plasticity of intrinsic excitability has been reported in numerous regions of the brain, including hippocampus, cerebellum and neocortex (see for reviews Daoudal and Debanne, 2003; Zhang and Linden, 2003) the question arises to what extent proteolytic activity in extracellular space could support these processes? In the following sections, we discuss literature related to proteolysis-mediated and activity-dependent changes in neuronal excitability and E-S potentiation (see also Figure 1).

With regard to rapid actions, several studies indicate that thrombin, trypsin and their protease activated receptors (PAR-1 and PAR-2) may play a crucial role in shaping neuronal excitability. Activation of PARs is initiated by site-specific proteolytic cleavage in the N-terminal extracellular region, which uncovers a tethered ligand activating Gα q/11, Gα i/o, or Gα12/13-proteins and results in activation of multiple intracellular signaling pathways depending on the activating ligand (reviewed in Ben Shimon et al., 2015, this Special Issue). Interestingly, thrombin and it’s receptor PAR-1 were shown to mediate pathway specific effects on neuronal excitability. For instance, during EEG recording *in vivo*, thrombin was shown to rapidly enhance neuronal excitability as early as 2–4 min following application (Lee et al., 1997). In another study, exogenous thrombin application rapidly (within 10 min) decreased inhibitory currents and increased CA3 pyramidal neurons spontaneous action potentials discharges, while in CA1, it produced enhanced inhibitory input (Maggio et al., 2013). Such site specific acute actions of thrombin were ascribed to enhanced expression of PAR-1 in these regions (Maggio et al., 2013). Interestingly, thrombin application or PAR-1 activation produced rapid increase in population spike that was more pronounced than accompanying EPSP enhancement, an effect that saturated the ability of the tissue to undergo tetanus-induced LTP (Maggio et al., 2008). In addition, thrombin lowered the threshold for generating epileptic seizures in CA3 region of the hippocampus (Maggio et al., 2008). Also in dentate gyrus neurons, PAR-1 activation resulted in rapid membrane depolarization within 10–20 s and increased spiking of dentate granule cells (Han et al., 2011). Thus, the role of PAR-1 receptors seems to be of particular importance in rapid modulation of neuronal excitability of hippocampal neurons (see also Figure 1). It should be stressed, that PAR1 activation in astrocytes can trigger release of glutamate into the extracellular space and activate neuronal NMDA receptors, further supporting membrane depolarization (Lee et al., 2007). Thus, by releasing glutamate and D-serine, a single astrocyte can affect, in a synchronous manner, neuronal plasticity in many thousands of nearby excitatory synapses by activating NMDARs (Henneberger et al., 2010). Moreover, activation of PAR-1 by thrombin mediates induction of MMP-9 expression (Choi et al., 2008), a metalloprotease crucial for neuronal plasticity, learning and memory (Huntley, 2012). Altogether, it seems that PAR1-mediated signaling may be even more complex and interaction between proteases, PARs and neuronal excitability will require more detailed studies.

Another member of the family of receptor for proteases activated by trypsin, namely PAR-2, was previously implicated in rapid protease-dependent alteration of neuronal excitability and epileptic activity *in vivo* (Lohman et al., 2008). Notably, acute activation of PAR-2 resulted in significant membrane depolarization and depression of neuronal spiking in cultured hippocampal neurons (Gan et al., 2011). In another study, rapid PAR-2 activation via SLIGRL peptide resulted in reduced trypsin release and decreased afterdischarges within electroencephalograms (EEG) following electrically evoked kindling in amygdala as early as 10 min following peptide application (Lohman et al., 2008). Interestingly, activation of PAR-2 with trypsin reduced N-type voltage-gated Ca^2+^ current and PAR-2 agonists reduced action potential firing frequency in rat peripheral sympathetic nerve neurons (Kim et al., 2014). In conclusion, these studies indicate that PAR-2 activation reduces neuronal excitability and therefore has an anti-epileptogenic role. However, most recently, PARs were also implicated in regulation of neuronal excitability through modulation of ion channels function and opposite conclusions were drawn. In bronchopulmonary sensory neurons, activation of PAR-2 for 2 min with activating peptide reduced large conductance Ca^2+^-activated potassium channel (BK) current, which resulted in increased excitability (Moss et al., 2015). Thus, it is plausible that activation of PAR-2 in various neurons results in different functional outcomes and therefore it remains to be further established *in vivo* whether PAR-2 has an anti-seizure and antiepileptogenic role (see also Figure 1).

Not only thrombin and trypsin were shown to rapidly modulate neuronal spiking. Another protease neuropsin, a trypsin-like serine protease strongly expressed within hippocampus (Chen et al., 1995) was shown to be crucial for hippocampal dependent learning and synaptic LTP (Tamura et al., 2006). In support of this view, exogenous application of recombinant neuropsin lasting several minutes potentiated EPSPs and to even a larger extent also population spike for whole duration of recording (3 h, Tamura et al., 2006) further documenting that extracellular proteases may trigger fairly rapid and persistent E-S potentiation. Unfortunately, while the mechanism of synaptic plasticity was investigated, the mechanism of E-S potentiation in this system remains to be established. However, in another study from that group, neuropsin knockout mice exhibited significantly reduced inhibitory inputs from parvalbumin interneurons among many other abnormalities (Hirata et al., 2001), suggesting that excitation/inhibition balance could be under control of neuropsin (see also section below).

The above mentioned findings suggest that proteases may differentially affect synaptic and spike components. In our most recent study, we addressed the role of MMP-3 and MMP-2/9 activity in supporting E-S potentiation in the CA3 region of the hippocampus (Wójtowicz and Mozrzymas, 2014). We found that acute MMP-3 inhibition with specific inhibitor NNGH impaired EPSP and spike potentiation as early as 5 min following LTP induction with tetanic stimulation or pairing of mossy fiber synapses activity with CA3-CA3 synapses. In contrast, the effect of MMP-2/9 inhibition was visible not earlier than 1.5 h post LTP induction (Wójtowicz and Mozrzymas, 2014). Thus, MMP-3 and MMP-2/9 may differentially shape expression of E-S potentiation in the CA3 region of the hippocampus. Most strikingly, the reduction in spike potentiation following MMP-3 inhibition was more pronounced than would be expected from impaired synaptic plasticity alone, suggesting that synaptic and nonsynaptic components are regulated separately. Of note, MMP-3 activity regulates availability of several proteins known to support long-term plasticity, such as NMDARs, brain-derived neurotrophic factor (BDNF), cell adhesion molecules and other MMPs including MMP-9 (reviewed in Van Hove et al., 2012). Thus, properly balanced MMP-3 activity may be permissive for expression of E-S coupling. The exact mechanism underlying such MMP-3 impact on E-S potentiation remains elusive. However, we may suggest at least two possible explanations. First, we found that LTP of NMDARs function is lacking upon application of MMP inhibitors (Wójtowicz and Mozrzymas, 2014). Therefore, taken the great importance of Ca^2+^ flux mediated by NMDARs for E-S potentiation (see above), MMP-activity could support E-S potentiation by supporting NMDARs function. Second, since MMP-3 cleaves immunoglobulin-like cell adhesion molecule 5 (ICAM-5, Conant et al., 2010) and recently, a soluble N-terminal fragment of ICAM-5 was shown to rapidly (10–15 min) induce neuronal spiking in dose and time-dependent manner (Niedringhaus et al., 2012) we can speculate that MMP-3 could cleave ICAM-5 and support E-S potentiation by providing N-terminal fragment of this immunoglobulin like protein.

Finally, when considering acute action of proteases on neuronal E-S coupling, it should be born in mind that in most cases, data were obtained in the *in vitro* models, where proteases were applied in excess to the entire preparation and timing of such administration could not be very precise. To give an example, it was reported that neuropsin at concentrations below 1.8 mU/ml potentiated EPSPs and population spike amplitude to similar extent (Tamura et al., 2006). However, doubling this concentration resulted in a significant depression of synaptic transmission (Tamura et al., 2006). In another study, protease plasmin applied for 6 h at 100 nM impaired the maintenance of synaptic LTP (Nakagami et al., 2000). However, in another study plasmin applied at the same concentration (100 nM) for 10 min was shown to equally potentiate EPSPs and population spikes following patterns of stimuli inducing short-term potentiation (Mizutani et al., 1996) which typically promote neuronal plasticity, but not E-S potentiation. Thus, it needs to be emphasized that in physiological conditions, the enzyme concentration, site of action and timing are likely to be of crucial importance.

### Proteolytic Regulation of Ion Channels and Membrane Excitability

Intracellular proteolytic processing and regulation of voltage-gated calcium channels via the carboxy-terminal domain is well documented (for a review, see Catterall, 2011). However, studies relating directly rapid extracellular proteolysis with function of voltage- or ligand gated channels with respect to neuronal spiking are sparse. Recent studies suggest that a complex interaction occurs between certain extracellular proteases and function of voltage-gated channels. In particular, a number of voltage-gated ion channels contribute to spike afterdepolarization (ADP). ADP is mediated largely by persistent sodium current (I_NaP_), which is opposed by outward current mediated by KCNQ/M potassium channels (I_M_) and has a crucial impact on neuron ability to generate multiple spikes. Notably, in young (postnatal day 6–15) rats, exogenously applied thrombin was shown to increase neuronal excitability in just several minutes in an NMDAR-independent manner. In addition, exposure of CA3 pyramidal neurons to exogenous thrombin for 10 min was shown to result in less negative membrane potential (depolarization by 2 mV) and it produced a hyperpolarizing shift of voltage dependence of tetrodotoxin-sensitive persistent voltage-gated sodium channel activation which resulted in more pronounced I_NaP_ current at negative potentials (Isaeva et al., 2012). Thus, in addition to action on PAR-1, thrombin may promote neuronal excitability via regulation of certain ion channels.

From the point of view of neuronal plasticity, ligand-gated ion channels providing Ca^2+^ are indispensable. NMDA-receptors are known to play a pivotal role in neuronal plasticity, learning and acquisition of spatial reference memory (Malenka and Bear, 2004; Nakazawa et al., 2004). Therefore possible interaction of extracellular proteolysis with NMDARs function has been addressed in several studies. Importantly, as stated above, Ca^2+^ flux mediated by NMDARs remains necessary for expression of E-S potentiation (Wójtowicz and Mozrzymas, 2014).

It appears that some proteases released following enhanced neuronal activity could potentially regulate NMDAR-currents. For example, matrix metalloprotease (MMP-7, but not MMP-2/9) was shown to cleave GluN1 subunit of NMDARs and suppress Ca^2+^ current (Szklarczyk et al., 2008) while MMP-9 proteolysis was implicated in lateral mobility of these receptors (Michaluk et al., 2009). In addition, another matrix metalloprotease (MMP-3) was shown to cleave NMDARs at glycine binding site, but the functional consequences of this cleavage remain to be determined (Pauly et al., 2008). Neuropsin knockout mice exhibited significantly smaller NMDAR-mediated currents in principal neurons of the basal amygdala (Attwood et al., 2011). However in the hippocampus, direct application of neuropsin did not modulate NMDAR-mediated current (Komai et al., 2000). Another protease tPA was reported to cleave the GluN1 subunit of the NMDA receptor, but in the case of this protease such cleavage enhanced NMDAR function (Nicole et al., 2001). However, the proteolytic degradation of NMDARs was questioned in other *in vitro* studies were high doses of tPA were used (Liu et al., 2004; Pawlak et al., 2005). In particular, nonproteolytic effect of tPA on NR2B-containing NMDA receptors was reported (Pawlak et al., 2005). Therefore, it remains to be established *in vivo* to what extent neuropsin or tPA could rapidly modulate NMDAR function following enhanced synaptic activity.

In a study from our laboratory, the impact of MMP-9 on NMDAR currents was tested in cultured neurons. We showed that recombinant MMP-9 increased NMDAR desensitization and shortened the decay time constant of evoked NMDAR-mediated current in cultured neurons *in vitro* (Gorkiewicz et al., 2009). Altogether, the prevalent view from these studies is that excess of proteases activity decrease NMDAR-mediated currents. This contrasts with the general view that proteolytic activity supports NMDAR-dependent synaptic LTP. Second, with regard to MMP, in our study, we found that LTP of NMDARs function is lacking upon application of MMP inhibitors (Wójtowicz and Mozrzymas, 2014). Thus, MMP-activity is necessary to support rather than suppress NMDARs function. Therefore, it is not clear to what extent proteases could directly affect NMDAR function *in vivo*.

We have recently showed that the permanent deprivation but also excess of protease MMP-9 activity negatively affect LTP expression in MF–CA3 and Sch-CA1 hippocampal projections (Wiera et al., 2013, 2015). Thus, in the hippocampus, epileptiform activity involves enhanced excitation among pyramidal cells and excessive NMDA receptor activation (Miles and Wong, 1987; Meier et al., 1992) which is associated with extensive synaptogenesis and recurrent collaterals and reduced synaptic inhibition (Dingledine et al., 1986; Merlin and Wong, 1993). Several lines of evidence indicate that extracellular proteolysis mediated by trypsin, plasmin or thrombin and several other enzymes could be involved in these processes (Yamada and Bilkey, 1993; Mizutani et al., 1996; Lee et al., 1997). Indeed, overexpression of serine protease inhibitor 1 (Nexin-1, PN-1) resulted in enhanced GABAergic and glutamatergic transmission, increased polyspiking and enhanced NMDAR function resulting in enhanced LTP (Lüthi et al., 1997). Thus, while synaptic transmission seems to be differentially regulated by PN-1 activity, PN-1 manipulation always resulted in enhanced neuronal excitability. Moreover, mutant mice lacking tPA, or plasminogen, are resistant to seizure induction and neuronal cell death after kainic acid administration (Tsirka et al., 1995, 1997). Thus, modulation of NMDARs function most likely represents a fine-tuning related to specific time windows and loci of the proteolytic action which is dependent on the neuronal network activity. Indeed, considering a key role of NMDARs in secretion of several proteases (Michaluk et al., 2007), one can expect some feedback mechanisms in which, following enhanced synaptic activity and activation of NMDARs, released proteases regulate NMDARs function and thereby Ca^2+^ entry affecting synaptic plasticity which, in turn, affects further release of proteases.

Finally, some evidence document more general role of proteolysis in regulation of ion channels beyond hippocampus. For instance, an α-secretase ADAM10 was shown to cleave ectodomain of β2 subunit of voltage-gated sodium channel in Chinese hamster ovary cells (Kim et al., 2005). However, to what extent ADAM10 protease could affect function of this channel remains to be elucidated. Moreover, as mentioned in above section, rapid modulation of N-type voltage-gated Ca^2+^ channels and BK channel function occurs following PAR-2 activation in sensory or peripheral nerves *in vitro* (Kim et al., 2014; Moss et al., 2015). Interestingly, MMPs 2/9 were shown to directly alter the gating properties and function of retinal cyclic nucleotide-gated channel in concentration-dependent manner by proteolysis of extracellular domain of the receptor (Meighan et al., 2012, 2013). It is also worth mentioning, that in nonneuronal cells, epithelial sodium channel (ENaC) was shown to be regulated by serine proteases (Rossier and Stutts, 2009), cysteine protease cathepsin-S (Haerteis et al., 2012) or metalloprotease meprin β (Garcia-Caballero et al., 2011).

Thus, it is important to realize that besides modulation of key receptors involved in synaptic plasticity, rapid extracellular proteolysis may also affect the function of voltage-gated ion channels. However, since some of above mentioned studies were carried out *in vitro*, future *in vivo* studies are necessary to verify this interesting possibility. Since voltage gated sodium channels have recently been implicated not only in regulating membrane excitability, but in addition to that in adhesion, migration, path finding and transcription (Brackenbury and Isom, 2008), interaction between extracellular proteases and voltage-gated sodium channels most likely goes beyond regulation of neuronal excitability.

### Long-Term Effects of Extracellular Proteolysis on Neuronal Excitability

While above mentioned studies addressed acute actions of extracellular proteolysis, several studies in which knockout or overexpression of protease genes have further indicated, that prolonged deficit or excess of extracellular proteolysis may have a crucial impact on neuronal excitability and E-S potentiation. For instance, in neuropsin mutant mice, increased susceptibility for hyperexcitability (polyspiking) was reported in response to repetitive afferent stimulation with no deficits in hippocampal synaptic LTP (Davies et al., 2001). This suggests that neuropsin is a protease supporting both synaptic plasticity and plasticity of neuronal excitability. In the amygdala, PAR-1 was proposed to promote contrasting neuronal responses depending on the emotional status of an animal by a dynamic shift between distinct G protein-coupling partners. In particular, in PAR-1 knockout mice, basal amygdala pyramidal neurons exhibited enhanced firing rate 48 h following fear conditioning when compared to wild type controls (Bourgognon et al., 2012). Similar results were obtained in naïve mice that were bilaterally infused with SCH79797, a PAR-1 function inhibitor before above mentioned conditioning paradigm (Bourgognon et al., 2012). Altogether, PAR-1 may be involved in regulation of neuronal excitability in amygdala in experience-dependent manner.

Complementary conclusions were drawn by manipulating PN-1. This endogenous serine proteases inhibitor is provided by glia and neurons and affects activity of thrombin, tPA, plasmin, trypsin and several other serine proteases in ECM (Lüthi et al., 1997). In agreement with studies discussed above, animals lacking and overexpressing PN-1 exhibited severe changes in EPSP-spike coupling in the hippocampus. Notably, mice knocked out in PN-1 gene exhibited increased susceptibility to kainic acid-induced seizures, characteristic polyspiking, while at the same time they exhibited decreased NMDA/AMPA ratio and no changes in basal EPSCs or IPSCs (Lüthi et al., 1997). Altogether, these data indicate that the proper balance between serine proteases and metalloproteases and their endogenous inhibitors (serpin inhibitors, tissue inhibitors of metalloproteases TIMPs) in long-term window are strongly involved in control of neuronal excitability.

With regard to extracellular proteolysis, most recent studies indicate that regulation of neuronal intrinsic excitability might rely on increased proteolytic cleavage of certain ion channel subunits. First, membrane bound protease BACE1 (beta-site APP cleaving enzyme 1, memapsin 2, Asp 1) expressed in hippocampal dentate gyrus, hilus and stratum lucidum (Laird et al., 2005) was shown to be crucial for function of sodium and potassium channels. Nav1.1 and 1.3 channels are mainly located at somatodendritic regions, while Nav1.2 and 1.6 are distributed to axons (Lai and Jan, 2006). In rat primary cortical cultures, prolonged (2 h) exposure to BACE1 resulted in cleavage of β2 subunit of sodium voltage-gated ion channels (Nav1.1). Moreover, overexpression of BACE1 reduced sodium-current *in vitro* indicating that BACE1 activity regulates cell-surface sodium channel function (Kim et al., 2007). Consequently, neurons from BACE1 knockouts exhibited overexpression of voltage gated sodium channels (Nav1.1, 1.2, 1.6), had decreased firing threshold, larger population spikes and developed spontaneous epileptic seizures (Hu et al., 2010), but exhibited unaltered LTP, LTD or NMDARs function (Laird et al., 2005). Interestingly, in a most recent study, BACE1 was shown to regulate neuronal excitability through an nonenzymatic interaction with KCNQ channels mediating I_M_ current. Notably, BACE1^−/−^ hyperexcitability was explained by loss of repolarizing I_M_-current (Hessler et al., 2015). Potassium channels mediating I_M_-current do not participate appreciably in action potential repolarization due to their slow kinetics. However, this current is crucial during AHP of medium duration (Gu et al., 2005; Tzingounis and Nicoll, 2008) and shapes spike frequency adaptation. In addition, I_M_-current is also present in axons and presynaptic terminals, where it modulates firing patterns and transmitter release (Martire et al., 2004; Vervaeke et al., 2006; Sun and Kapur, 2012; Battefeld et al., 2014). Thus, BACE1 seems to play an important role in neuronal excitability and is essential for cognitive, emotional, and synaptic functions (Laird et al., 2005) and BACE1 inhibitors may normalize membrane excitability in Alzheimer’s disease patients with elevated BACE1 activity (Kim et al., 2007).

### Impact of Extracellular Proteolysis on Inhibitory Transmission and Excitation/Inhibition Balance

As mentioned in previous chapters, change in the balance of excitation/inhibition is considered to be a powerful mechanism gating information flow in neuronal networks. Therefore altered GABAergic inhibition is expected to significantly affect the outcome of dendritic integration and ultimately the information flow. Moreover, inhibition of GABA_A_Rs facilitates the induction of synaptic plasticity (Stelzer et al., 1994). With this regard, exogenous thrombin application rapidly (less than 1 min) and robustly (80%) decreased inhibitory currents in cultured neurons (Hashimotodani et al., 2011). Consequently, acute application of thrombin was shown to lower the threshold for generating epileptic seizures and to change spontaneous activity of CA3 hippocampal pyramidal cells through activation of PAR-1 (Maggio et al., 2008). Indeed, in acute brain slices, thrombin rapidly (within 10 min) decreased inhibitory currents and increased CA3 pyramidal neurons spontaneous action potentials discharges (however in CA1, it produced rather enhanced inhibitory input, Maggio et al., 2013). Exogenously applied plasmin was also shown to quickly (1 min) reduce GABAergic currents in CA1 pyramidal neurons (Mizutani et al., 1997).

In several transgenic models, GABAergic transmission was modulated following long-term manipulation of proteolytic activity in extracellular milieu. For instance neuropsin KO mice exhibited increased propensity to hyperexcitability (polyspiking) in response to repetitive afferent stimulation. These animals were additionally more prone to seizure activity on kainic acid administration and heightened immediate early gene (c-fos) expression throughout the brain (Davies et al., 2001). Importantly, neuropsin mutant mice displayed normal hippocampal LTP and exhibited no deficits in spatial navigation tasks (Davies et al., 2001) implying that neuropsin may regulate neuronal spiking to larger extent than synaptic plasticity (see also below). Given that most neuropsin is stored in the extracellular space as a nonactive proform and that neuropsin activity occurs rapidly following neuronal activity (5 min, Matsumoto-Miyai et al., 2003) it is expected that local neuropsin-mediated proteolysis could significantly change the E-S coupling. Indeed, loss of neuropsin was implicated in decreased efficiency of somatic inhibitory input, reduced synchronization of pyramidal cells and ultimately impaired long-term plasticity of glutamatergic transmission (Tamura et al., 2012).

Mice overexpressing the protease tPA showed an enhanced LTP and exogenous application of tPA enhanced L-LTP in rat hippocampal slices (Baranes et al., 1998; Madani et al., 1999). Thus, tPA proteolysis remains important for synaptic LTP. In a seminal paper, Frey and coworkers investigated the role of protease tPA in E-S plasticity (Frey et al., 1996). In acute brain slices from tPA knockout mice they described increased feed forward inhibition and E-S depression, lack of E-S shift following LTP induction and reduced paired pulse spike facilitation. By using GABA_A_R-antagonist bicuculline, they found much more pronounced population spike increase without any impact on EPSPs. Thus, tPA differentially supports EPSP and spike plasticity and most likely GABAergic system is involved in these processes.

We also studied the role of GABAergic system in mediating effects of MMPs activity on E-S potentiation in CA3 hippocampal region (Wójtowicz and Mozrzymas, 2014). We found that by blocking GABA_A_Rs with low, nonsaturating doses of picrotoxin, population spike in CA3 region of the hippocampus increased four-fold in the absence of any change in synaptic drive (Wójtowicz and Mozrzymas, 2014; Supplementary Figure 1) confirming a crucial role of somatic inhibitory input in braking neuronal firing. However, we still observed effects of MMP inhibition on E-S potentiation in the presence of GABA_A_Rs blocker, indicating that at least in our system, effects associated with MMPs inhibition are not primarily related to GABAergic input.

In the last decade, the regulation of GABAergic transmission via endocannabinoid-mediated retrograde signaling were intensely investigated (Heifets and Castillo, 2009; Kano et al., 2009). Following postsynaptic membrane depolarization, release of endocannabinoids can retrogradely activate presynaptic receptors (CB1) and suppress GABA release in inhibitory synapses, a phenomenon called depolarization induced suppression of inhibition (DSI; Wilson and Nicoll, 2001; Wilson et al., 2001). It has been recently shown *in vivo* that CA1 hippocampal place cell firing, following injection of waking patterns of CA1 place cells discharge recorded during a spatial task resulted in endocannabinoid-mediated decrease of GABAergic transmission through CB1Rs (Dubruc et al., 2013). This resulted in enhanced firing probability and better spike precision compared to situation of no DSI (Dubruc et al., 2013). It has been postulated that such improved spike-time precision could play a role in both spike-timing coordination and network oscillations in the hippocampus (Dubruc et al., 2013). It is expected that modulation of endocannabinoid signaling may have a profound impact on neuronal input-output function and eventually information processing. With this regard, recently, activation of neuronal protease activated receptor (PAR-1) has been shown to affect this system. Notably, in cultured hippocampal neuron *in vitro*, activation of neuronal PAR-1 via thrombin or specific activating peptide was shown to trigger retrograde signaling mediated by endocannabinoid 2-AG and presynaptic CB1 receptors, ultimately resulting in massive suppression of inhibitory synapses (Hashimotodani et al., 2011). Taken that activation of astrocytic PAR-1 results in potentiation of excitatory transmission (Lee et al., 2007; Mannaioni et al., 2008) it is expected that acute activation of PAR-1 (e.g., by thrombin) may result in enhanced EPSP-spike coupling via suppression of somatic inhibition and enhanced synaptic excitation.

In conclusion, it appears that both acute and long-term manipulations in proteases activity may affect effectiveness of inhibitory drive and neuronal excitability.

### Proteolytic Degradation of Extracellular Matrix and Neuronal Excitability

Among many substrates, proteases released to extracellular space cleave ECM components, yielding cryptic peptides and providing space for structural rearrangements of neuronal compartments. The molecular mechanism underlying interaction of ECM components and neuronal excitability remains elusive. It appears however, that proteolytic processing of some components of ECM or adhesion molecules following enhanced neuronal activity may affect neuronal excitability by affecting function of certain ionotropic channels.

Recently, hyaluronic acid was shown to directly gate L-type voltage gated channels in hippocampal CA1 neurons and subsequently regulate postsynaptic Ca^2+^ entry, synaptic plasticity and learning and memory (Kochlamazashvili et al., 2010). While these effects were observed in excitatory synapses, it is interesting to consider more broad regulation of neuronal function by ECM rigidity. Integrity of specialized ECM areas surrounding neuronal cell bodies, perineuronal nets (PNNs) were shown to be crucial for hippocampal-dependent learning and memory (Gogolla et al., 2009; Kochlamazashvili et al., 2010; reviewed in Wang and Fawcett, 2012). PNNs were shown to envelop Kv3.1b expressing neurons and it has been suggested that these structures likely support the high firing frequencies of fast-spiking interneurons (Hartig et al., 1999). Indeed, *in vitro* enzymatic treatment of PNNs with chondroitinase ABC which resulted in degradation of glycosaminoglycans (e.g., chondroitin sulfate and hyaluronan) was shown to result in increased excitability and spike firing in parvalbumin positive interneurons *in vitro* but not in pyramidal cells (Dityatev et al., 2007). In another *in vivo* study, exogenous application of chondroitinase in the ventral hippocampus resulted in a selective increase in dopaminergic neurons and pyramidal cells firing rate, as determined a week later (Shah and Lodge, 2013). Long term treatment (9 days long) of neuronal cultures with hyaluronidase transformed the normal network firing burst into oscillations and epileptiform-like activity (Vedunova et al., 2013). However, whether this increased neuronal firing was induced by enhanced synaptic drive or changes in intrinsic neuronal excitability was not investigated.

On the other hand, ectodomains of some cell adhesion molecules are cleaved by several secreted (e.g., MMPs) or membrane bound (e.g., ADAM10) proteases and released into extracellular space following neuronal activity (e.g., nectin-1, L1 and NCAM, ICAM-5; Thelen et al., 2002; Hinkle et al., 2006; Lim et al., 2012; Niedringhaus et al., 2012) and may additionally regulate neuronal firing. In particular, MMP-3 was shown to cleave ICAM-5 following enhanced neuronal activity and LTP (Conant et al., 2010). Soluble N-terminal fragment of this immunoglobulin like protein was shown to induce neuronal spiking in dose and time-dependent manner through integrin β1 signaling (Niedringhaus et al., 2012). Thus, a cascade involving secreted proteases and the products of rapid proteolysis containing RGD motif could rapidly affect neuronal function through integrin signaling.

The importance of ECM rigidity in neuronal excitability is additionally evident in several knockout models. For instance, tenascin-R knockout mice exhibited hyperexcitability in the CA1 region but were not susceptible to pilocarpine induced seizures (Brenneke et al., 2004). Tenascin-R knockout mice exhibited decreased somatic GABAergic inhibition due to altered GABA_B_-mediated transmission, as well as increased basal excitatory synaptic transmission and impaired NMDAR-mediated LTP (Saghatelyan et al., 2001; Brenneke et al., 2004; Bukalo et al., 2007). Of note, tenascins can bind to neuronal sodium channels and play an important role in regulation of sodium channel density at axon initial segments (AISs) and nodes of Ranvier (Srinivasan et al., 1998). This further emphasizes that components of ECM and PNNs may not solely build a scaffold for neurons, but play an important yet subtle role in regulation of neuronal excitability and E-S coupling.

Finally, proteins secreted in latent form and activated by proteolysis in the extracellular environment may add another level of complexity to the input-output functions of neurons. One prominent example involves BDNF. This protein is secreted in neuron activity-dependent manner (Hartmann et al., 2001; Gärtner and Staiger, 2002) and it’s availability in extracellular space is regulated by proteolytic processing involving e.g., tPA-mediated activation of plasmin (Seidah et al., 1996) and MMP-9 (Mizoguchi et al., 2011). BDNF is transported anterogradely and retrogradely and can activate TrkB receptors both pre- and postsynaptically. Importantly, the association of BDNF with TrkB affects ion channels including voltage gated sodium (e.g., Nav1.9), potassium (e.g., Kv1.3, Kir3), calcium channels as well as NMDARs and AMPARs within a range of seconds to minutes through intracellular signaling cascades (Cunha et al., 2010). While the impact of BDNF and its receptor TrkB on synaptic plasticity has been extensively studied (for a review, see Cunha et al., 2010) it’s role in neuronal excitability has received less attention. In one study BDNF was shown to acutely potentiate population spike more than would result from EPSP amplification alone (Messaoudi et al., 2002). Acute BDNF application resulted in significantly enhanced E-S coupling indicating that neuronal excitability is not linearly affected by EPSP boost (Messaoudi et al., 2002). Consequently, BDNF is localized and upregulated in areas implicated in epileptogenesis (reviewed in Binder et al., 2001). Altogether, indirectly, by regulating availability of BDNF and other secreted proteins, proteolytic activity in ECM may serve a potentially important role in shaping E-S potentiation, but verification of this possibility requires further studies.

### Regulation of Neuronal Output by Axon Initial Segment

The AIS is a specialized unmyelinated part of the axon involved in the initiation of action potentials. This region is enriched in several types of voltage gated sodium (Na_V_), potassium K^+^ (K_V_1), Ca^2+^ channels and specific molecular complexes such as adhesion proteins (CAMs), ECM and cytoskeleton adaptors (Clark et al., 2009). However, similar to dendrites, AIS does not seem to simply passively generate and convey action potentials. In a recent study using fluorescence recovery after photobleaching (FRAP), recovery of K_V_2.1 channels cluster within AIS occurred rapidly with a time constant of approximately 11 s (Sarmiere et al., 2008). This suggests mobility of ion channels is not restricted by AIS. In addition, functional studies showed that variable position of AIS within the axon produces significant differences in neuronal excitability (Kress et al., 2010). Notably, Na_V_1.6 channels distributed more proximally and exhibited lower overall density in dentate granule cells than CA3 pyramidal cells and that resulted in higher voltage threshold of dentate granule neurons (Kress et al., 2010). In addition, recent studies have showned a novel level of regulation of neuronal excitability in response to excitatory and inhibitory inputs which relies on changes in length and molecular composition of AIS (Grubb and Burrone, 2010; Kuba et al., 2010). Assembly of the AIS requires interactions between scaffolding molecules and voltage-gated sodium channels, but the molecular mechanisms that stabilize the AIS remain unclear. Members of chondroitin sulfate proteoglycans have been found to surround AIS such as aggrecan, brevican (John et al., 2006), neurocan, versican, tenascin-R (Bruckner et al., 2006). Consequently, recent studies have highlighted the role of the ECM degrading enzymes including matrix metalloproteinases, serine proteases (thrombin and the urokinase plasminogen activator system) and cysteine proteases in AIS stabilization. For instance, tenascin-C and -R were implicated in regulation of action potential initiation due to impact of this ECM component on density of sodium channels (Srinivasan et al., 1998). Tenascin-C additionally plays an important role in modulating L-type voltage gated calcium channels (Evers et al., 2002; Kochlamazashvili et al., 2010) while tenascin-R integrity may be essential for proper function of perisomatic GABAergic inhibition. Notably, tenascin-R knockout mice showed reduction of such GABAergic inhibition provided to CA1 neurons by basket cells (Bukalo et al., 2007).

In mature nervous system, AIS is surrounded by a dense ECM of unique composition, including the chondroitin sulfate proteoglycan brevican which shows preferred expression within AIS (John et al., 2006). Brevican is degraded by matrix metalloproteinases and aggrecanase-1 (ADAMTS4; Nakamura et al., 2000) and it’s interaction with CAMs such as neurofascin isoform NF-186 is essential for molecular assembly of the AIS (Hedstrom et al., 2007; Zonta et al., 2011). Similarly, since L1cam protein interacts with ankyrins which couple voltage-gated sodium channels to the spectrin-based membrane skeleton (Srinivasan et al., 1988), cleavage of L1cam by neuropsin following enhanced neuronal activity was proposed to result in dynamic rearrangement of clustered membrane proteins (Shiosaka, 2004) such as sodium channels. In summary, it seems plausible that rapid proteolytic processing of some constituents of ECM and cell adhesion molecules may affect stability of AIS. However, it is currently not clear what is the time scale of this process and functional studies addressing neuronal firing following modulation of AIS stability following proteolysis are lacking.

## Discussion

Plasticity of local or global neuronal excitability can have profound consequences for neuronal input-output function and paradoxically also for synaptic plasticity for at least two reasons. First, changes in intrinsic properties of a part of a dendrite will directly affect local summation of EPSPs (reviewed in Beck and Yaari, 2008). Second, the amplitude of bAPs in dendrites is an important factor for LTP induction in hippocampal neurons and local synaptic LTP is associated with enhanced local membrane excitability which affects other synapses in the vicinity of the activated synapses (reviewed in Johnston et al., 2003). Therefore by affecting local dendritic Ca^2+^ spikes bAPs may additionally boost synaptic plasticity in respective parts of the dendrite (reviewed in Frick and Johnston, 2005). Finally, the Ca^2+^ influx that accompanies the bAPs or synaptic activity during the LTP induction protocols may also regulate the expression and function of dendritic ion channels. Ultimately, global changes in neuronal intrinsic excitability are expected to be critically important in entraining other neurons within hippocampal networks into a synchronized population discharge (Traub and Wong, 1982). However, the underlying mechanism is not fully understood.

We are just beginning to unravel the importance of intrinsic changes in physiology of the central nervous system accompanying neural plasticity, learning and memory. Importantly, changes in intrinsic neuronal properties are increasingly being recognized as an important, pathophysiologically relevant aspect of psychiatric and neurological disorders. In a recent review, intrinsic plasticity of nucleus accumbens medium spiny neurons excitability has been proposed to be an important player in development of addiction (Kourrich et al., 2015). Thus, drugs such as cocaine may affect several voltage gated conductances and the outcome of such modulation may also contribute to the shaping of the addiction phenotype (Kourrich et al., 2015). In addition, recent advances indicate that some disorders of the central nervous system, such as chronic pain and epilepsy, are associated with significant alterations of intrinsic properties of neurons (reviewed in Beck and Yaari, 2008). In particular, increased intrinsic dendritic excitability has been found to underlie disorders in mouse models of chronic epilepsy and Alzheimer disease (reviewed in Beck and Yaari, 2008). Cognitive decline in Alzheimer disease was also previously proposed to be a result of destabilization of Ca^2+^ homeostasis and modulation of intrinsic excitability (Disterhoft and Oh, 2006; Santos et al., 2010; Kaczorowski et al., 2011).

In this review, we highlighted several mechanisms by which proteases released into extracellular space following enhanced neuronal activity may rapidly participate in shaping neuronal input-output function in various neuronal compartments, not limited to synaptic loci (Figure 1). Studies discussed above and our most recent study addressing the role of MMPs in E-S potentiation (Wójtowicz and Mozrzymas, 2014) emphasize that extracellular proteolysis may be capable of fine-tuning information flow and storage by affecting targets beyond synapses. Thus, in our view, this opens an interesting field for future research, addressing the role of rapid and long-term extracellular proteolysis on important aspects of information processing in neurons, such as propagation of synaptic input to soma, initiation of action potential, spike frequency adaptation, properties of action potential and dendritic backpropagation. In particular, we are lacking functional studies addressing activity-dependent proteolytic modulation of dendritic or somatic channel function.

## Conflict of Interest Statement

The authors declare that the research was conducted in the absence of any commercial or financial relationships that could be construed as a potential conflict of interest.
